# Cancer information seeking and scanning behavior among Nepalese migrants in Japan and its association with preventive behavior

**DOI:** 10.1371/journal.pone.0235275

**Published:** 2020-06-29

**Authors:** Divya Bhandari, Akihiko Ozaki, Yurie Kobashi, Asaka Higuchi, Prakash Shakya, Tetsuya Tanimoto

**Affiliations:** 1 Medical Governance Research Institute, Tokyo, Japan; 2 Department of Breast Cancer, Jyoban Hospital of Tokiwa Foundation, Iwaki, Japan; 3 Department of Public Health, Fukushima Medical University, Fukushima, Japan; 4 Department of Community and Global Health, Graduate School of Medicine, The University of Tokyo, Tokyo, Japan; Makerere University, School of Public Health, UGANDA

## Abstract

**Background:**

Increasing attention is being paid to cancer information seeking (CISE) (active searching for cancer-related health information) and information scanning (CISC) (passive collection of cancer-related health information) among migrants. However, information is lacking with respect to the extent and distribution of CISE and CISC among migrants, particularly in Japan. This study aimed to evaluate the prevalence of both CISE and CISC, to clarify factors associated with CISE and CISC, and to elucidate the association of CISE and CISC with basic cancer knowledge and preventive behavior among Nepalese migrants living in Tokyo, Japan.

**Methods:**

Nepalese migrants living in Tokyo were recruited from March to August 2019, with snowball sampling. We collected data on CISE, CISC, sociodemographic components, health-related factors, knowledge about risk factors for cancer, and cancer-prevention behavior using a structured questionnaire. We employed several regression approaches to fulfill our study objectives.

**Results:**

Out of the total 200 participants, 53 (27%) were actively involved in CISE and 176 (88%) in CISC. Internet was the most common information source. High education level and Japanese language skills were positively associated with both CISE and CISC. Migrants with low perceived health status were more likely to perform CISC, while those who had been ill last year and who perceived proper access to doctors were more likely to undertake CISE. Migrants with high CISE (B = 0.10, 95% CI: 0.01, 0.19) and high CISC (B = 0.16, 95% CI: 0.08, 0.23) were more likely to have better knowledge on risk factors of cancer. Furthermore, migrants with high CISE were more likely to eat fruits (B = 0.17, 95%CI: 0.01, 0.32), undergo pap smear test (OR = 1.72, 95%CI: 1.12, 2.65), and colonoscopy (OR = 6.02, 95%CI: 1.63, 22.13).

**Conclusion:**

In this study, the proportion of Nepalese migrants who deliberately undertook CISE was low, while the practice of CISC was relatively common. Given that the CISE was associated with cancer-prevention behavior, proper strategies should be implemented to alleviate barriers for CISE and improve its impact on providing reliable evidence about cancer to migrants in Japan.

## Background

Cancer diagnosis used to be widely believed as a death sentence but that is no longer the case. As the world population is growing and aging, the number of cancer deaths is increasing, with nearly half of all cancer deaths being among people aged 70 or older. Based on the population-based cancer registry, in Nepal, lung cancer is major cancer in males while breast cancer is leading cancer in females [1]. In Japan, one out of every two Japanese now develops cancer, with stomach cancer being the leading form, followed by a colon, liver, and pancreatic [2]. As a consequence, the three core goals of the Japan Cancer Society are to:

1. promote cancer screening
2. disseminate correct knowledge/raise awareness of cancer
3. care for cancer patients and survivors [3].

The cancer burden is increasing in almost every country yet around 40% of cancer cases could be prevented by reducing exposure to cancer risk factors. Nevertheless, cancer remains an increasing global health problem, with an estimated 18.1 million incidences and 9.6 million deaths in 2018 [4]. Although cancer leads to premature mortality, physical disability, mental stress, and financial crisis [5–7], it can often be prevented by following a healthy lifestyle [8]. Around one-third of cancer deaths are due to the five leading behavioral and dietary risks: high body mass index, low fruit and vegetable intake, lack of physical activity, tobacco use, and alcohol use. Some 30–50% of cancers can be prevented by avoiding risk factors and implementing existing evidence-based prevention strategies. Even after the appearance of cancer, adverse health effects can be alleviated by early diagnosis and timely treatment [9]. In this respect, knowledge of cancer-related health information, including its consequences, risk factors, preventive strategies, and treatments is important, as such knowledge can improve disease management skills, including the ability to make informed medical decisions, adopt healthy lifestyles, and better adjust to the extremely stressful situations that follow on from diagnosis of the condition [10,11].

Methods of collecting cancer-related health information can be categorized into cancer information seeking (CISE) and scanning (CISC) [12]. CISE is an active search of information, and it has been traditionally a focus of the research in this study domain [13]. In contrast, CISC is an unintentional or inactive way of collecting cancer information [13], and it has been recently suggested to be a more natural way of cancer information collection. Both CISE and CISC are crucial to gain insights into various topics, including cancer-related risk factors, preventive behavior to decrease cancer incidence, and effective screening and treatment to cope with cancer-related challenges [14]. However, multiple demographic, psychological, environmental, cultural, and financial factors act as barriers to access health information, such as lack of education, low income, poor health status, limited access to doctors, lack of health insurance, and fear of the disease [15–17].

Given these backgrounds, migrants could be a population who struggle to be properly engaged in both CISE and CISC. The trend of migration from less developed to developed countries has become more common, and usually migrating people come from poor socioeconomic conditions in their home countries. Following relocation, migrants further experience additional challenges related to living and working conditions, such as lack of social networks, emotional stress adapting to the new environment, work overload, and high living costs [18–20]. Collecting health information might be difficult due to a variety of factors, including a lack of knowledge about medical rules and regulations in the host country [21–23]. As a result, migrants might give less priority to healthy behavior and compromise their dietary habits, exercise pattern, health screening, and health-information seeking behavior [24–26]. These could put them at risk of developing cancer and delay cancer detection.

Japan has become a popular destination for migrants from Asia. Japan has witnessed a recent change in migrant policy following workforce shortages due to a decrease in the working-age population and an increase in the elderly population. The number of immigrants is expected to increase significantly over the coming years. Over the last decade, the number of Nepalese migrants living in Japan increased more than 7-fold, reaching a total of 88,951 in 2018. In 2018 alone, Nepalese migrants increased by 11% [27]. Most of them have student and dependent’s visa status, often working part-time in convenience stores, fast food shops, or factories. Others work in Indo-Nepalese restaurants and very few occupy jobs as skilled workers in Japanese companies. The majority of the Nepalese students studying Japanese have to work long hours, often exceeding legally permitted limits. Long working hours coupled with unhealthy dietary patterns and lifestyle put them at a higher risk of developing diseases like cancer. Unlike Nepal, Japan has advanced treatment and diagnostic/screening services for cancer. Some of the screening services are even free of cost in some cities. Many Japanese undergo routine full-body health check-ups, which include identifying cancer markers, which are usually free for the elderly. However, migrants need to be well informed of their options in order to utilize available health facilities effectively. In Japan, most of the information (general and medical) is provided in Japanese. Therefore, many migrants might have difficulties understanding information clearly, particularly medical information, which requires high language proficiency and biological or medical knowledge to comprehend fully and unambiguously. This raises questions about how they can meet their suitable and appropriate health information needs, especially for life-threatening diseases like cancer. Therefore, it is crucial to understand the CISE and CISS needs and behavior of migrants.

Despite the growing number of migrants and the increasing burden of cancer worldwide, little research has been carried out to understand the CISE and CISC of migrants. To the best of our knowledge, no study has been conducted on this issue among international migrants in Japan. This study aims to identify the prevalence of CISE and CISC behavior, factors associated with the CISE and CISC behavior, and association of CISE and CISC with the knowledge of risk factors towards cancer and disease-prevention behavior.

## Methods

### Settings and participants

A cross-sectional study was conducted from March to August 2019 among Nepalese migrants living in Tokyo. This study recruited Nepalese migrants aged 18 years and above, living in Tokyo for more than six months, and who were able to read and write in the Nepalese language. We recruited study participants from different parts of Tokyo using snowball sampling. Researchers, along with one Nepalese community resource person (health background) from Tokyo, were involved in the data collection. Additional support was provided by other Japanese citizens and Nepalese migrants. We first disseminated information about the research among Nepalese migrants with the help of Nepalese and Japanese local persons. Their available time and feasible place were confirmed beforehand and we collected data visiting different Nepalese restaurants, shops, and apartments. Besides that, some data were collected from a health counseling camp conducted on March 21^st^, 2019, at the Everest International School Ogikubo, Tokyo, and also through another health counseling session conducted in Hachioji, Tokyo on June 16^th^, 2019. Only participants who agreed to participate were included in this study. Before data collection, migrants were informed about the study objectives using a prepared information sheet. Only participants who agreed to participate after reading the information sheet were asked to fill the survey. Participation was voluntary, and migrants were allowed to withdraw at any time if they felt uncomfortable. Confidentiality was maintained.

### Survey sheets

A self-administered structured validated questionnaire was adopted for data collection by the authors after reviewing previous studies on CISE and CISC [28–30]. The questionnaire, unavailable in Nepalese was translated, back translated, and further reviewed by oncologists and public health professionals. A pretest was done among 15 Nepalese migrants. Some Nepalese translated words which were difficult to understand were revised after pretest before final data collection. The survey sheet questionnaire, in the Nepalese language comprised five sections: 1) socio-demographic characteristics, 2) health-related factors, 3) cancer information collection (CISC and CISE), 4) knowledge of risk factors of cancer, 5) disease preventive behavior related to cancer.

### Variables

1) Sociodemographic characteristics. Sociodemographic variables included age, gender, length of stay in Japan, visa status (student, dependent, cook, or other working visas), marital status, educational status, Japanese language skills, and health insurance coverage. Regarding Japanese language skills, participants were asked to rate their skill from ‘very good to none’ (5-point items). They were further asked if they needed a language interpreter during any medical consultation (yes or no). Similarly, with regards to health insurance, which is mandatory in Japan, we asked two questions: ‘Do you have your Japanese health insurance card (Hokensho)? (yes or no). If the response was ‘yes’, they were further asked ‘If yes, how often do you pay the premium of the health insurance? (monthly or bi-monthly, not paid for 3–6 months, not paid for 6–12 months, not paid for more than a year).
2) Health-related factors. Health-related factors included self-rated health, “In general, how do you rate your current general health status?” (five-point items from ‘very good to very poor’). We also asked about personal and family history of cancer (yes or no), their actual health condition, ‘Have you been ill/had health problems in the past 12 months?’ (yes or no), accessibility to doctors, ‘Currently, do you think you have proper access to a doctor/health worker in Japan?’ (yes or no), the first place to seek help, ‘Which is the first place you go if you became ill?’(hospital, clinic, public health center, local pharmacies, home treatment only or other), consultation with a doctor, ‘Have you visited a doctor/health worker for medical consultation in the past 12 months?’(yes or no), numbers of consultations in the past 12 months and any problems faced during a consultation.
3) CISE and CISC behavior. For measuring CISE behavior, we used two questions: ‘Thinking about the past 12 months, did you actively look for information about cancer from a doctor or other people or the media?’(yes/no). If the response was ‘yes’, they were further asked ‘If yes, were you actively looking for information about cancer in the past 12 months from any of the following sources (television, radio, newspapers, magazines/posters, internet, health professionals, family and friends)’. Responses were measured using 5-point Likert items from ‘never (scored 0) to always (scored 4)’ for each source. All the seven items were summed up to calculate total scores (minimum 0 to maximum 28), where high total scores indicated CISE behavior.

For the assessment of CISC behavior, we similarly used the two questions: ‘Thinking about the past 12 months, did you hear or come across information about cancer from a doctor, other people or from media, even when you were not actively looking for it’ (yes/no). If the response was ‘yes’ they were further asked ‘If yes, how often did you hear or receive cancer information indirectly from each of the following sources?’ (television, radio, newspapers, magazines/posters, internet, health professionals, family, and friends). Responses were measured using 5-point Likert items from ‘never (scored 0) to always (scored 4)’ for each source. All seven items were summed up to calculate total scores (minimum 0 to maximum 28), where high total scores indicated high CISC behavior.

4) Knowledge about risk factors of cancer. For the assessment of knowledge about risk factors of cancer, we used a validated Nepalese questionnaire used in a previous study [30]. The questionnaire included 10 items, and responses were measured by ‘yes,’ ‘no’, and ‘don’t know’. Correct responses were rated as 1, and incorrect and ‘don’t know’ responses were rated 0. All the items were summed up to calculate total scores and a high score indicated high knowledge about risk factors for cancer.
5) Cancer prevention behavior. In the assessment of preventive behavior towards cancer, we prepared questions concerning tobacco smoking, drinking alcohol, diet, physical exercise, and undergoing cancer screening as follows; responses for smoking were rated as 1 for ‘yes’, 0 for ‘no’, and ‘used to smoke before but not now’. Similarly, for alcohol drinking responses were rated as 1 for ‘yes’, 0 for ‘no’. In addition to this question, the frequency of drinking was also requested (no alcohol or less than one alcoholic drink/day, 1 drink/day, 2 drinks/day, 3 drinks and more/day, or question not applicable). For diet, migrants were asked the number of days they eat fruits/vegetables in a week and the number of servings they ate on that particular day. Total intake (separately for vegetable and fruits) was calculated by adding the number of days and number of servings, and thus total intake was used for analysis. Regarding physical exercise, questions were asked about time (number of days in the month and hours on that particular day) and the type of exercise. However, after data collection, we found that the responses written by participants for types of exercise and respective durations were not appropriate for categorization. Different types of exercises (moderate and vigorous intensity) were mixed up together and timing for each type of exercise was also not clear. This prevented us from accurately categorizing exercise intensity based on the recommended World Health Organization guideline. WHO states different time durations for rigorous and mild intensity. Therefore, the analysis of exercise was done based on yes/ no response only. Concerning cancer screening programs, four specific types were investigated: mammography within the last two years, colonoscopy within the last 10 years, PSA within the last 2 years, and having a Pap smear within the last 3 years.

### Data analysis

Collected data were entered in Epi Data version 3.1. The entered data was exported to Stata version 13.1(College Station, Texas, USA) for data analysis. We performed three analyses in this study. First, descriptive statistics were conducted to present the quantitative description of the sociodemographic, health condition, CISE and CISC, knowledge of risk factors of cancer, and preventive behavior related to cancer.

Second, we assessed the factors associated with CISE and CISC. For this, univariate analysis was conducted using simple linear regression to identify the significant factors. Then, multiple linear regression was conducted for adjusting confounders and covariates. A separate analysis was conducted for both CISE and CISC. We considered all sociodemographic and health-related factors, using the backward stepwise variable selection method (p > 0.05). While assessing factors associated with seeking, scanning was also included as exposure variables and vice versa. Variables having categories with a small number of participants were regrouped as appropriate. All variables were checked for collinearity using ‘vif’ (variation inflation factor) and ‘collin’ commands in Stata. Variables having vif more than 10 were excluded from the analysis. The distribution of the residuals was checked for normality.

Finally, we assessed the association of CISE and CISC with knowledge and preventive behavior (dietary, exercise and screening) related to cancer using a multiple linear regression model for continuous outcome variables (knowledge, drinking habits, vegetables, and fruits intake) and multiple logistic regression for dichotomous outcome variables (smoking, screening, and exercise practices). For each of the regression analyses, we considered all sociodemographic and health-related factors, using the backward stepwise variable selection method (p > 0.05); CISE and CISC were treated as main exposure variables. With regard to screening, we only calculated regression analysis for a pap smear test (females aged 20 or above) and colonoscopy (all participants aged 40 or above). However, analyses for mammography and PSA tests were not conducted due to the limited sample size of the eligible population. Statistical significance was set at 0.05.

### Ethical considerations

This study obtained approval from the Research Ethics Committee of the Medical Governance Research Institute (MG2018-15), Tokyo, Japan. Before data collection, an information sheet was given to the participants to read and that information was also provided verbally. It included the details of the research including the purpose and procedure of research, type/nature of questions in the questionnaire, potential benefits and risks, and measures to maintain the anonymity of the participants and confidentiality of the responses. They were also informed that their participation in this study is voluntary. They were free not to participate at all or to withdraw from the study at any time, without any penalty. Once the participants agreed to fill the survey by saying ok, participants’ ID was written and the questionnaire was provided. All the participants in this study gave verbal informed consent. The questionnaire was filled by participants themself.

## Results

A total of 200 samples were included in the analysis. Out of 210 collected samples, ten were excluded because of missing data. Missing data and data inconsistency were examined before analysis.

Table 1 summarizes the socio-demographic characteristics of the migrants included in this study. The median age of migrants was 31 years (range: 20–71 years) and median years of stay in Japan was four years (range:1–27 years). More than half (79%) of the migrants were married, while 46%% had completed an undergraduate or post-graduate degree. Almost all (96%) had health insurance (which is mandatory for all citizens and those living legally in Japan, including all immigrants) with 62% paying their premiums monthly or bi-monthly. Of the total, 45% rated their Japanese language skills as ‘average’, while more than half (55%) mentioned they needed a Japanese interpreter when visiting a medical center/hospital.

**Table 1:** Socio-demographic characteristics of participants (N = 200).

Socio-demographic variables	n	%
**Age** [median (range)]	31 (20–71)	
**Gender**		
Female	76	38.0
Male	124	62.0
**Mean years of stay in Japan** [median (range)]	4 (1yr–27yrs)	
**Marital status**		
Married	158	79.0
Unmarried	42	21.0
**Visa status**		
Student visa	47	23.5
Dependent visa	54	27.0
Cook visa	35	17.5
Other working visa	49	24.5
Other status	15	7.5
**Education**		
Primary/Secondary	45	22.5
Higher secondary	63	31.5
Bachelor	66	33.0
Master and above	26	13.0
**Have health insurance**		
Yes	192	96.0
No	8	4.0
**How often do you pay health insurance**		
Paid regularly every 1 or 2 months	123	61.5
Not paid for 3–6 months	43	21.5
Not paid for 6–12 months	15	7.5
Not paid for more than a year	11	5.5
**Need a Japanese language interpreter when visiting a clinic/hospital**		
Yes	109	54.5
No	91	45.5
**Japanese language skill**		
Very good/good	60	30.0
Average	90	45.0
Not good/none	50	25.0

Table 2 illustrates the health-related characteristics of the participants. Out of 200 migrants, 102 believed that they had proper access to doctors and health professionals in Japan. The hospital was the first place to seek help for about 51%, followed by a clinic (41%) and a local pharmacy (4%). Out of 74 migrants who suffered from health problems in the previous 12 months, 69 sought a health consultation with a doctor. Around 31% of migrants mentioned that they faced difficulties in seeking medical consultation in Japan. Of the total, 50% of migrants thought that their health status was ‘fair’, while only 3% perceived themselves as being in poor health. Although there was no acknowledged personal history of cancer among any participants, 7% of them identified a family history of cancer.

**Table 2:** Health-related characteristics of participants (N = 200).

Variables	n	%
**Proper access to doctor and health professional in Japan**		
Yes	102	51.0
No	98	49.0
**Ever been to a clinic or a hospital in Japan for a checkup**		
Yes	133	66.5
No	67	33.5
**Been ill or had health problems in the past 12 months**		
Yes	74	37.0
No	126	63.0
**Consultation with a doctor in the past 12 months**		
Yes	69	34.5
No	131	65.5
**The first place to seek help when you became ill**		
Clinic	82	41.0
Local pharmacy	8	4.0
Hospital	101	50.5
Public health center	7	3.5
Home treatment	2	1.0
**Faced problem while having medical consultation in Japan**		
Yes	61	30.5
No	139	69.5
**Perceived health status**		
Very good	17	8.5
Good	76	38.0
Fair	99	49.5
Poor/Very poor	8	4.0
**Family history of cancer**		
Yes	14	7.0
No	186	93.0

Table 3 demonstrates cancer knowledge, preventive behaviors, and cancer information collection among the participants. The mean score of knowledge was 5.9 (SD 2.8) on a scale of 1 to 10. Out of the total,176 (88%) were involved in CISC, while only 53 (27%) were engaged in CISE. Around 25% were involved in both seeking and scanning behavior. Among the 200 migrants, 43% had a habit of drinking alcohol, and 22% used to smoke. On average, the migrants usually ate vegetables and fruits 4 days/week with 1.6 servings of vegetables and 1 serving of fruit per day. Of the total, only 38 (19%) engaged in physical exercise and 29 (15%) had undergone cancer screening.

**Table 3:** Cancer knowledge, preventive behaviors and cancer information collection among participants (N = 200).

Variables	n	%
**Knowledge about cancer risk factors** (Range 1–10) [mean(sd)]	5.9 (2.76)	
**Smoking tobacco**		
Yes	44	22.0
No	156	78.0
**Drinking alcohol**		
Yes	86	43.0
No	114	57.0
**The average amount of alcoholic drinks intake per day**		
no drink per day	29	14.5
1 drink per day	26	13.0
2 drinks per day	17	8.5
3 or more drinks per day	16	8.0
**Fruits intake** [mean(sd)]		
How many days in a week do you eat fruits	4.3 (2.0)	
How many servings of fruits in those days	1.5 (0.6)	
**Vegetables intake** [mean(sd)]		
How many days do you eat vegetables in a week	4.3 (1.9)	
How many servings of vegetables in those days	1.6 (0.7)	
**Physical exercise**	**n**	**%**
Yes	38	19.0
No	162	81.0
**Cancer Screening**		
Yes	29	14.5
No	171	85.5
**Performed cancer screening**		
Mammography within last 2 years	8	4.0
Colonoscopy within last 10 years	18	9.0
PSA within the last 2 years	9	4.5
Pap-smear test within the last 3 years	9	4.5
**Cancer information seeking and scanning behavior**		
**Cancer information seeking**		
Yes	53	26.5
No	147	73.5
**Cancer information scanning**		
Yes	176	88.0
No	24	12.0
**Practice of both seeking and scanning**	51	25.5

**Fig 1:**
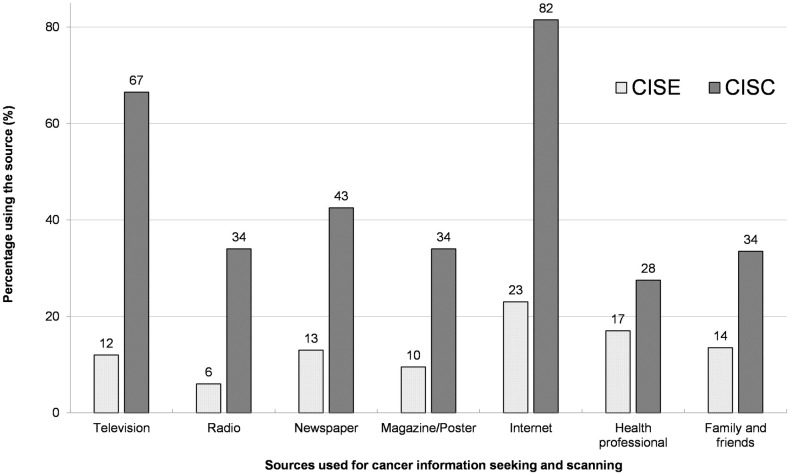
Different sources used for cancer information seeking and scanning (N = 200).

Fig 1 represents the sources used for CISE and CISC. The Internet was the most used source for both CISE and CISC, where 41.0% (82/200) and 11.5% (23/200) used it for CISC and CISE, respectively. Similarly, the least used sources for CISC were radio, magazines/posters, and friends/family (used by 34 migrants). For CISE, the least used source was radio (used by only four migrants).

Table 4 demonstrates multiple linear regression analyses of the factors associated with the CISE and CISC. After adjusting for covariates and confounders, it was seen that migrants with higher education (master level or higher) were more likely to undergo CISE (B = 3.08, 95% CI: 1.01, 5.15) and CISC (B = 3.28, 95% CI: 1.74, 6.85) compared to those with primary level education. Migrants who had good Japanese language skills were more likely to undergo both CISE (B = 1.31, 95% CI: 0.74, 2.54) and CISC (B = 2.35, 95% CI: 0.61, 4.09) compared to those with poor language skill. Migrants who had been ill during the previous 12 months (B = 1.60, 95% CI: 0.24, 2.96) and who believed they could access doctor/medical professionals in Japan (B = 1.22, 95% CI: 0.35, 2.09) were more likely to seek cancer information actively; migrants who perceived their health status was very good were less likely to undergo CISC (B = -5.17, 95% CI: -9.42, -0.93) compared to those in poor health. Migrants who had undergone cancer information seeking were more likely to scan cancer information (B = 0.25, 95% CI: 0.04, 0.47).

**Table 4:** Factors associated with cancer information seeking and scanning in the last 12 months among Nepalese migrants in Japan.

	CISE		CISC	
Variables	Coefficient	CI	Coefficient	CI
Age	-0.028	(-0.08 0.02)	-0.072	(-0.15 0.002)
Education level				
Primary/Secondary	Base			
Higher secondary level	-0.99	(-2.03 0.05)	1.43	(-0.47 3.34)
Bachelor level	0.42	(-0.77 1.63)	2.09*	(0.10 4.07)
Masters level or higher	3.08**	(1.01 5.15)	3.28**	(1.74 6.85)
Japanese language skill				
Not good	Base			
Average	-0.13	(-1.10 0.83)	0.16	(-1.47 1.74)
Well	1.31*	(0.74 2.54)	2.35**	(0.61 4.09)
Proper access to doctor/medical professional				
No	Base			
Yes	1.22**	(0.35 2.09)	-0.24	(-1.64 1.14)
Been ill in the past 12 months				
No	Base			
Yes	1.6*	(0.24 2.96)	0.64	(-1.02 2.31)
Perceived health status				
Poor	Base			
Fair	2.83	(-0.34 5.33)	-4.67*	(-8.73–0.59)
Good	2.24	(-0.34 4.82)	-5.79**	(-9.78–1.80)
Very good	1.86	(-0.18 5.90)	-5.17*	(-9.42–0.93)
CISC	0.12	(-0.001 0.24)		
CISE			0.25*	(0.04 0.47)

*p<0.05,

**p<0.01,

***p<0.001.

Abbreviations: CI, Confidence Interval; CISE, Cancer information seeking; CISC, Cancer information scanning; Adjusted for ethnicity, gender, visa status, marital status, insurance status, family history of cancer, perceived health status

Table 5 illustrates the association of the CISE and CISC with knowledge and preventive behavior. After adjusting for confounders and covariates, migrants who had undergone CISE (B = 0.10, 95% CI: 0.01, 0.19) and CISC (B = 0.16, 95% CI: 0.08, 0.23) were more likely to have knowledge of cancer risk factors. While migrants with high CISC were less likely to drink alcohol (B = -0.05, 95% CI: -0.09, -0.01), migrants with high CISE were more likely to eat fruits (B = 0.17, 95% CI: 0.01, 0.32). Similarly, with high CISE behavior, migrants were more likely to undergo a pap smear (OR = 1.72, 95% CI: 1.12, 2.65), and colonoscopy (OR = 6.02, 95% CI: 1.63, 22.13).

**Table 5:** Association of knowledge and preventive behavior with cancer information seeking and scanning: Multiple regressions (linear and logistic) (N = 200).

	CISE		CISC	
Outcome variables ^a^	Coefficient	CI	Coefficient	CI
Knowledge about risk factor of cancer	B = 0.10*	(0.01 0.19)	B = 0.16***	(0.08 0.23)
Lifestyle				
Drinking alcohol	B = 0.04	(-0.01 0.09)	B = -0.05*	(-0.09–0.01)
Smoking tobacco	OR = 0.88	(0.75 1.04)	OR = 1.05	(0.95 1.15)
Eating fruits	B = 0.17*	(0.01 0.32)	B = 0.05	(-0.12 0.13)
Eating vegetables	B = -0.11	(-0.41 0.19)	B = -0.04	(-0.16 0.09)
Exercise	OR = 0.98	(0.87 1.12)	OR = 0.98	(0.88 1.09)
Screening				
Colonoscopy (n = 42)	OR = 6.02**	(1.63 22.13)	OR = 1.60	(0.89 2.87)
Pap test (n = 76)	OR = 1.72*	(1.12 2.65)	OR = 1.04	(0.84 1.30)

Table 5 presents results of multiple linear regression (unstandardized coefficient B) or logistic regression (OR) predicting each health outcome from seeking and scanning after controlling for other relevant factors

*p<0.05,

**p<0.01,

***p<0.001.

Abbreviations: OR, Odd ratio; CI, Confidence Interval

^a^ Adjusted for age, gender, ethnicity, visa status, marital status, insurance status, access to a doctor in japan, family history of cancer, perceived health status, consultation with doctors.

## Discussion

In this study, we primarily found that a small proportion of Nepalese migrants in Japan were engaged in CISE, while a majority of them were involved in CISC. Migrants who underwent CISE and CISC were more likely to have knowledge of cancer risk factors. However, migrants were more likely to adopt cancer-preventive behaviors (like eating fruits and undergoing some form of screening), only when they actively seek cancer information (CISE).

In our study, only 26% of the migrants were actively involved in CISE, although a large proportion of them underwent CISC. This finding is quite similar to previous studies where the CISE was comparatively lower than CISC [29]. However, this similarly should be interpreted cautiously, given that cancer predominantly affects the middle-aged and elderly population, and that the previous study only considered those aged 40 or above. In contrast, in our study, a large proportion of the participants were below 40, with their median age being 31. It should also be noted that an association between age and general health-information seeking behavior has been found to vary in earlier reports [31–33]. Thus, there is a need for analysis of factors that may have caused a similarity or discrepancy between previous studies and ours.

The low prevalence of CISE in this study is based on findings of the regression analyses. In our study, migrants with low Japanese language skills were less likely to undertake CISE. The language barrier is considered to be one of the main problems for migrants in acquiring information in a previous study also [34]. If an individual is unable to understand the language of the host country, he/she cannot collect information and utilize medical facilities despite having wishes to do so. In our study, nearly half of the migrants (49%) believed they do not have proper access to medical doctors/ health workers in Japan, and this was negatively associated with their CISE. Usually perceived low accessibility or increased uncertainly might further motivate a person to seek information. However, our finding is quite the opposite, this could be because to seek information for a specific disease like cancer, people need to be extra mindful or they should be pursued or suggested to do so. Proper access to doctors can enhance insight on the importance and methods of cancer information acquisition and further encourage migrants to seek it [35,36]. Although Japan has advanced and world-recognized medical services, as well as universal health coverage, it is not easy to find English-speaking medical clinics/hospitals in Japan. This means that there may be a major confounding factor, in that migrants have physical access to medical staff but are unable to communicate with them, resulting in them stating that they do not have access as a result of a language barrier.

Migrants who had been ill within the previous 12 months were more likely to undertake CISE. It can be said that a collection of health information may be a common behavioral response among those facing health challenges [37]. However, it was interesting to find that these migrants were more likely to collect health information, not on their disease, but a different disease like cancer after they suffered from a non-cancer health problem. This may be because experiences of health challenges might have enhanced their baseline interest in health information. Furthermore, their contact with health workers might encourage them to undergo CISE. Similarly, as shown in previous studies, we found that a low education level was also associated with low CISE [38]. Education could enable people to better access, understand, and communicate health information which could explain this finding.

In our study, only the CISE was associated with adopting cancer-prevention behavior like eating fruits and undergoing specific cancer screening (Pap smear test and colonoscopy), although both CISE and CISC were found to be associated with higher knowledge of cancer risk factors. This could be because the participants who were engaged in CISE would have more proactive attitudes toward adopting preventive behavior. CISE results in empowering people to understand the potential benefit of adopting specific behavior more clearly and thus encourages them to adopt healthy lifestyle components. This finding highlights the importance of CISE and the need to promote it among migrants. However, in a previous study, both CISE and CISC were significantly associated with the adoption of cancer-prevention behavior [28], which is inconsistent with our findings. This may have been because the participants of the previous study could have had a larger baseline interest in cancer and its prevention than those of our study because all of them were aged 40 or above.

Regarding the cancer information source, the Internet was the most popular source for both CISE and CISC in this study. This is natural, considering that it is difficult to obtain cancer-related health information in Japan in their native language in media other than the Internet. This is also true for migrants other than Nepalese and for Nepalese in other countries [39]. In this respect, there is a great potential for the Internet to serve as an important channel for cancer-related information dissemination among migrants, and the Internet can play a huge role in facilitating CISE behavior while addressing the underlying problems at the same time. Nonetheless, in the age of fake news, health information obtained from unauthorize and unofficial online sources may not reliable and appropriate [40]. Furthermore, highly technical language and a wide range of information could be overwhelming for people with low education levels. Therefore, the use of other authorized reliable sources should be encouraged, along with the Internet to ensure the reliability and accuracy of the health information. In addition, future investigations are necessary on digital devices used to access the Internet, languages to be used, and availability and affordability of the Internet.

Although CISE was associated with the adoption of some cancer-prevention behavior, some behavior, like exercise and not smoking tobacco were not associated with it in this study. This could be because the participants may have not been accustomed to rapid changes post-migration in terms of socio, cultural, economic aspects. Despite having cancer-related information, different underlying factors such as being busy, work pressure, and stress might have played a role in adopting such behavior [41]. Therefore, in-depth studies are needed to address and understand the hidden factors.

## Implication of this study

Given that the CISE was associated with both cancer knowledge and the adoption of cancer prevention behavior, awareness programs must be conducted to encourage migrants to engage in CISE. The Internet can be used as a source of cancer information dissemination for busy and hard-to-reach populations like migrants. However, safeguards would be needed to ensure that migrants have appropriate health literacy to discriminate between accurate and inaccurate sources. Furthermore, there is a need to help them obtain the information through other channels, not just the Internet. Central and municipal authorities have a lot of opportunities to improve on the creation and dissemination of important health information, including the types of screening programs available and how to access them. Most importantly, concerned authorities and organizations should make efforts to provide accurate and appropriate information, particularly all forms of health information and access to medical services in various languages. Professional translators can be employed in hospitals/medical centers to create a migrant-friendly environment. In addition, in-depth qualitative studies should be conducted among different nationalities and different age groups to better understand the quality of cancer information obtained by migrants and to identify all barriers and obstacles responsible for preventing them from fully understanding ways to prevent cancer and for monitoring their bodies to ensure that if cancer develops, it is found early, thus making it more likely to be treated successfully.

## Limitations and strengths of the study

The study has several limitations. First, we recruited our participants through convenience sampling, which might have led to selection bias and limited generalizability. However, convenience sampling is considered suitable for migrants, given the difficulty in reaching this population [42]. Second, responses were self-reported, and there was not any evidence to support the accuracy of their responses. Therefore, results might have response bias, resulting in over- or under-reporting of behavior. Third, although, we tried to measure the degree of the information obtained using a standard Likert scale, we could not measure the quality of the cancer information obtained by migrants. Due to the limited number of participants above age 40, we faced sample size issues while evaluating the association of CISE and CISC with several types of screening, including mammography and pap test. Furthermore, although we included potential confounders, there may well have been unidentified and unmeasured confounders that could have affected the participants; involvement in practicing cancer-prevention behavior.

Despite these limitations, this study delivers novel insights on CISE and CISC among the migrants studied. Further, to the best of our knowledge, this is the first study identifying the CISE, CISC, and cancer-prevention behavior among Nepalese migrants living in Japan. This study also provides information on the factors associated with CISE and CISC, and further, its association with knowledge and cancer-prevention practices among migrants living in Japan. Although conducted among the single nationality, these findings might also be relevant to other international migrants living in Japan. However, further study is warranted to elucidate whether our findings are applicable to immigrants from other countries.

## Conclusion

These results shed light on CISE and CISC among Nepalese migrants in Japan. The practice of CISE was relatively lower than CISC. The Internet was found to be the most common source for both. CISE and CISC were positively associated with the knowledge of cancer risk factors. However, migrants were more likely to adopt cancer-prevention practices (like eating fruits and undergoing cancer screening) only when they actively seek cancer information. This information could be used as a baseline to construct strategies to enhance cancer information collection among migrants, including and beyond Nepalese migrants, both in Japan and globally.

## Supporting information

S1 File Questionnaire (English). (PDF)

S2 File Questionnaire (Nepali). (PDF)
