# Supplementary material for: Cancer information seeking and scanning behavior among Nepalese migrants in Japan and its association with preventive behavior
Source: PLoS One. 2020 Jun 29;15(6):e0235275. doi: 10.1371/journal.pone.0235275 (PMC7347024; doi:10.1371/journal.pone.0235275)
Supplement: S1 File — (PDF) [file pone.0235275.s001.pdf]

Appendix: Questionnaire (English version)

Cancer information scanning and seeking behaviour among Nepalese migrants in Japan and its association with preventive behaviour

Participants ID# [ ] [ ] [ ]

Section 1: Socio-demographic characteristics

1) How old are you? …………… years old

2) What is your gender?

   [ ] Female [ ] Male

3) How long have you been in Japan? ………………years

4) What is your visa status?

   [ ] Student [ ] Dependent [ ] Cook Visa

   [ ] Other working visa [ ] Other, please specify……………………………..

5) What is your marital status?

   [ ] Married [ ] Unmarried [ ] Other, please specify………

6) Do you have your Japanese health insurance card (Hokensho)?

   [ ] Yes [ ] No

7) Do you pay the premium of the health insurance regularly?

   [ ] I pay monthly or once in two months

   [ ] Not paid since 3-6 months

   [ ] Not paid since 6-12 months

   [ ] Not paid for more than a year

8) What is your educational status?

   [ ] Illiterate/Non formal education

   [ ] Primary /Secondary level (1-10)

   [ ] Higher secondary level (10+2)

   [ ] Bachelor level

   [ ] Masters level or higher

9) Do you need a Japanese language interpreter when visiting a clinic/hospital?

[ ] Yes [ ] No

10) How well do you speak Japanese?

[ ] Very well [ ] Well [ ] Average [ ] Not well [ ] Not at all

Section 2: Health condition and health-seeking behavior

11) Currently, do you think you have proper access to a doctor/health worker in Japan?

[ ] Yes [ ] No

12) Have you ever been to Hospital/Clinic/Doctor/Health workers during your stay in Japan?

[ ] Yes [ ] No

13) Have you been ill/had health problems in the past 12 months?

[ ] Yes [ ] No

14) If yes, what was the health problem?...

15) Have you visited a doctor/health worker for medical consultation in the past 12 months?

[ ] Yes [ ] No

16) If yes, how many times did you seek medical consultation for health problems other than cancer?..................................times

17) If yes, how many times did you seek medical consultation related to cancer?......................times

18) Which is the first place you go if you become ill?

[ ] Clinic [ ] Public health center (Hokenjo)

[ ] Hospital [ ] Home treatment only

[ ] Local pharmacy [ ] Others(specify)………..

19) Did you face any problems while having seeking medical consultation in Japan?

[ ] Yes [ ] No

20) If yes, what kind of problems did you face?................................

21) In general, how do you rate your current general health status?

[ ] Very good [ ] Good [ ] Fair [ ] Poor [ ] Very poor

22) Do you have any family history of cancer?

[ ] Yes [ ] No

23) If yes, state the relationship and type of the cancer

i. State the relationship………………………………

ii. Type of cancer……………………………………..

24) Did you suffer from cancer in the past?

[ ] Yes [ ] No

25) If yes, please specify the type of cancer…………………………….

Section 3: Cancer information scanning and seeking behavior

26) Thinking about the past 12 months, did you actively look for information about cancer from doctors, from other people, or from the media?

[ ] Yes [ ] No

27) If yes, were you actively looking for information about cancer in the past 12 months from any of the following sources (check all that apply):

Source	Never	Rarely	Sometimes	Most of the times	Always
1 Television					
2 Radio					
3 Newspaper					
4 Magazine/Poster					
5 Internet					
6 Health professionals					
7 Family and Friends					

28) Thinking about the past 12 months, did you hear or come across information about cancer from doctors, from other people, or from the media even when you were not actively looking for it?

[ ] Yes [ ] No

29) If yes, how many times did you hear or come across information about cancer from each of the following sources when you were not actively looking for it. Please tick the correct options

Source	Never	Rarely	Sometimes	Most of the times	Always
8 Television					
9 Radio					
10 Newspaper					
11 Magazine/Poster					
12 Internet					
13 Health professionals					
14 Family and Friends					

Section 4: Awareness of risk factors of cancer

31 According to you, what are the risk factors of cancer?

Risk Factors	Yes	No	Don't Know
1 Smoking			
2 Excessive alcohol consumption			
3 High consumption of red meat			
4 Diet low in vegetables and fruits			
5 Physical inactivity			
6 Meat preserved by salts, nitrates, nitrites			
7 Infections			
8 Environmental pollution			
9 High exposure to radiation			
10 Family history of cancer			

Section 5: Preventive behaviour related to cancer

Smoking:

32 Do you currently smoke any tobacco products?

[ ] Yes [ ] No [ ] I used to smoke before but now I do not

Drinking:

33 Do you consume any alcoholic drink?

[ ] Yes [ ] No [ ] I used to drink before but now I do not

34 How frequently have you had an alcoholic drink?

[ ] less than 1 drink/day

[ ] 1 drink/day

[ ] 2 drinks/day

[ ] 3 or more drinks/day

[ ] Not applicable (This question is not applicable for me)

Diet:

35 In a typical week, on how many days do you eat fruit? .... numbers of days

36 How many servings of fruit do you eat on one of those days?.......................numbers of servings

37 In a typical week, on how many days do you eat vegetables?........................numbers of days

38 How many servings of vegetables do you eat on one of those days?..............numbers of servings

Physical Activities:

39 During the past 30 days, other than your regular job, did you participate in any physical activities?

[ ] Yes [ ] No

40 What kinds of physical activities or exercises do you participate in?...

41 During the past 30 days, how often have you been engaged in physical activities? …......…… days in total

42 During the past 30 days, about how long at each time did you spend in physical activities or exercises? ……………… mins/day

Screening:

43 Have you ever attended any type of cancer screening?

[ ] Yes [ ] No

44 Have you ever had any of the following screening?

Screening		Yes	No
1	Mammography within the last 2 years?		
2	Colonoscopy within last 10 years?		
3	PSA within the last 2 years?		
4	Pap –Smear test within the last 3 years?		

Thank you for your cooperation
