# Supplementary material for: Cancer information seeking and scanning behavior among Nepalese migrants in Japan and its association with preventive behavior
Source: PLoS One. 2020 Jun 29;15(6):e0235275. doi: 10.1371/journal.pone.0235275 (PMC7347024; doi:10.1371/journal.pone.0235275)
Supplement: S2 File — (PDF) [file pone.0235275.s002.pdf]

शिर्षक: जापानमा रहेका नेपाली प्रवासीहरुमा क्यान्सर सम्बन्धि जानकारीको खोजी गर्ने आचरण र त्यसको निवारक व्यवहारसंगको सम्बन्ध

उत्तरकर्ता (परिचयपत्र) संख्या ☐☐☐

कृपया सबै प्रश्नहरुको उत्तर दिनु होला। तपाईंलाई सबैभन्दा उपयुक्त लाग्ने उत्तरको नम्बरमा सहि ☑ चिन्ह लगाउनुहोला। "लेख्नुहोस "अथवा "खुलाउनुहोस" लेखिएको ठाउँमा चाहिं आफ्नो उत्तर प्रस्ट रुपमा लेख्नुहोला।

भाग १: सामाजिक तथा जनसांख्यिक जानकारी (विवरण)

१ तपाईको उमेर ………… वर्ष

२ लिङ्ग ☐1 महिला ☐ पुरुष ☐3 अन्य

३ तपाई जापान आउनुभएको कति वर्ष भयो ? ………… वर्ष

४ हाल जापानमा तपाईको भिसा स्ट्याटस कुन हो ?

☐1 विद्यार्थी भीसा ☐2 डिपेन्डेन्ट भीसा

☐3 कुक भीसा ☐4 अन्य वार्कीङ भीसा ☐5 अन्य (खुलाउनुहोस) ………

५. तपाईको वैवाहिक अवस्था के हो ?

☐1 विवाहित ☐2 अविवाहित ☐3 अन्य (खुलाउनुहोस) ………

६. के तपाईसग जापानको स्वास्थ्य विमा कार्ड(होकेनसो) छ ?

☐1 छ ☐2 छैन

७. तपाईले स्वास्थ्य विमाको प्रीमियम (किस्ता) नियमित रुपमा तिर्ने गर्नु भएको छ ?

☐1 प्रत्येक महिना वा दुई महिनामा तिर्ने गरेको छु ।

☐2 नतिरेको तीन देखि ६ महिना जति भयो ।

☐3 नतिरेको ६ देखि बाह महिना जति भयो ।

☐4 नतिरेको १ वर्ष भन्दा बढी भयो ।

८ तपाइको शैक्षिक स्तर कुन हो ?

1 निरक्षर / साक्षर (लेख्न पढन मात्र जान्ने)

2 प्राथमिक/ माध्यमिक (कक्षा १ देखि १० सम्म)

3 उच्च माध्यमिक (कक्षा ११ देखि बाह सम्म)

4 स्नातक (ब्याचलर लेभल सम्म)

5 स्नातकोत्तर र सो भन्दा बढी (मास्टर लेभल वा बढी)

९.के तपाईलाई जापानको अस्पताल/क्लिनिकमा स्वास्थ्य जाँच गराउँदा जापानीज भाषाको ट्रान्सलेटर (अनुवादक) आवश्यक पर्दछ ?

1 पर्दछ 2 पर्दैन

१० तपाई जापानीज भाषा कतिको रामोसँग बोल्न सक्नु हुन्छ ?

1 धेरै रामो 2 रामो 3 सामान्य

4 थोरै मात्रामा सक्छु 5 विल्कुलै सक्दिन

भाग दुई : स्वास्थ्य अवस्था र स्वास्थ्य सम्वन्धी जानकारी राख्ने व्यवहार

११. अहिलेको अवस्थामा जापानमा तपाईंको डाक्टर र स्वास्थकर्मीसँग पहुँच छ? (जस्तै जापानमा बिरामी पर्दा सजिलै गरि उपचार पाउन सक्नु)

1 छ 2 छैन

१२. तपाई जापान आएदेखि अहिले सम्म अस्पताल /क्लिनिक जानुभएको छ ?

1 छ 2 छैन

१३. विगत १२ महिनामा, तपाईलाई कुनै स्वास्थ्य सम्बन्धी समस्या भएको छ ?

1 छ 2 छैन

१४. यदि भएको छ भने, के समस्या भएको छ/थियो ?

....

....

....

१५. विगत १२ महिनामा, तपाईले कुनै डाक्टर / स्वास्थ्यकर्मीबाट स्वास्थ्य परामर्श लिनुभएको छ ?

1 छ 2 छैन

१६. यदि छ भने क्यान्सर सम्बन्धी परामर्श कति पटक लिनुभएको छ ?......................पटक

१७. क्यान्सर भन्दा अन्य स्वास्थ्य समस्याहरुको लागि तपाईले कति पटक परामर्श लिनुभएको छ ?.....................पटक

१८. तपाई बिरामी हुनुभएमा, तपाईले धेरैजसो जाँच गराउन जाने पहिलो स्थान कुन हो ?

1 क्लिनिक 2 औषधि पसल 3 अस्पताल

4 होकेन्जो (जनस्वास्थ्य केन्द) 5 घरेलु उपचार मात्र 6 अन्य खुलाउनुहोस ....

१९. तपाईले जापानमा उपचार गर्ने क्रममा केहि समस्या भोग्नुपरेको छ ?

1 छ 2 छैन

२० यदि छ भने, कस्तो खालको समस्या भोग्नु परेको छ ? खुलाउनुहोस ....

२१ सामान्यतया, तपाईले आफ्नो अहिलेको स्वास्थ्य स्थितिलाई कसरी मुल्याङकन गर्नुहुन्छ ?

1 धेरै रामो 2 रामो 3 सामान्य

4 नरामो 5 धेरै नरामो

२२ तपाईको परिवारमा अहिलेसम्म कसैलाई क्यान्सर भएको छ ?

1 छ 2 छैन

२३ यदि छ भने, कस्लाई भएको छ / थियो ?

नाता (खुलाउनुहोस): ....

कुन क्यान्सर भएको थियो: ....

२४ के तपाईलाई क्यान्सर भएको छ/ थियो ?

1 छ 2 छैन

२५ यदि भएको छ भने, के क्यान्सर भएको छ / थियो ? ....

भाग तीन : क्यान्सर सम्बन्धी जानकारीको खोजी गर्ने व्यवहार

२६. विगत १२ महिनाको अन्तरालमा, तपाईले **आफै सक्रिय भइ** क्यान्सर सम्बन्धी जानकारी डाक्टर, अन्य व्यक्ति, मिडियाबाट लिनुभएको छ या छैन ?

1 छ 2 छैन

२७. यदि छ भने, **आफै सक्रिय भएर** कतिपटक निम्न स्रोतद्वारा क्यान्सर सम्बन्धी जानकारी लिनुभएको छ। कृपया सहि विकल्पमा टिक गर्नुहोस।

स्रोत	कहिलेपनि लिएको छैन	मुस्किले कहिलेकाहि लिएको छ	कहिलेकाहि लिएको छु	प्राय लिएको छु	सधै लिएको छु
१ टिभी	1	2	3	4	5
२ रेडियो	1	2	3	4	5
३ पत्रपत्रिका	1	2	3	4	5
४ पोस्टर	1	2	3	4	5
५ ईन्टरनेट	1	2	3	4	5
६ स्वास्थ्यकर्मी	1	2	3	4	5
७ परिवार र साथीभाई	1	2	3	4	5

२८. विगत १२ महिनाको अन्तरालमा तपाईले **आफै सक्रिय रुपमा क्यान्सर सम्बन्धी जानकारी नलिएतापनि** तपाई क्यान्सरको बारेमा डाक्टर, अरु कुनै व्यक्ति, मिडियाबाट यसको बारेमा जानकारी पाउनु भएको छ ?

☐ छ ☐ छैन

२९ आफै **सक्रिय भएर क्यान्सर सम्बन्धी जानकारी नलिएतापनि** तपाईले कतिपटक निम्न स्रोतद्वारा क्यान्सर सम्बन्धी जानकारी पाउनुभएको छ। कृपया सहि विकल्पमा टिक गर्नुहोस

स्रोत	कहिलेपनि पाएको छैन	मुस्किले कहिलेकाहि पाएको छ	कहिलेकाहि पाएको छु	प्राय पाएको छु	सधै पाएको छु
१ टिभी	1	2	3	4	5
२ रेडियो	1	2	3	4	5
३ पत्रपत्रिका	1	2	3	4	5
४ पोस्टर	1	2	3	4	5
५ ईन्टरनेट	1	2	3	4	5
६ स्वास्थ्यकर्मी	1	2	3	4	5
७ परिवार र साथीभाई	1	2	3	4	5

३० तल दिएका विकल्पहरुमध्ये क्यान्सर निम्ताउने कारक तत्वहरु के-के हुनसक्छन ?

जोखिम कारकहरू	हो	होइन	थाहा छैन
क चुरोट सेवन	1	2	3
ख अत्याधिक रक्सी सेवन	1	2	3
ग धेरै रातो मासु खानाले	1	2	3
घ तरकारी तथा फलफुल कम सेवन	1	2	3
ङ कम शारीरिक गतिविधि	1	2	3
च रसायन राखेर लामो समय नबिगने गरी बनाएको मासु	1	2	3
छ, भाइरसको संकमण	1	2	3
ज वातावरण पदुर्षण	1	2	3
झ विकिरण	1	2	3
ञ परिवारमा कसैलाई क्यान्सर भएको अवस्था	1	2	3

भाग ५: क्यान्सर सम्बन्धि रोकथामका व्यवहारहरु

धुमपान

३१ के तपाईले चुरोट सेवन गर्नुहुन्छ ?

1 गर्छु 2 गर्दिन 3 पहिला गर्थे (अहिले छोडिसके)

३२ के तपाइले रक्सी पिउनुहुन्छ ?

1 पिउछु 2 पिउदिन 3 पिउथे अहिले पिउदिन

३३ यदि रक्सी पिउनुहुन्छ भने, दिनमा कतिपटक रक्सी पिउनुहुन्छ ?

1 १ गिलास भन्दा कम	2 दुई गिलास

3 १ गिलास	4 तीन गिलास भन्दा बढी

5 यो प्रश्न मलाई उपयुक्त छैन

आहारा

३४ साधारणतह, एक हप्तामा तपाई कति दिन फलफुल खानुहुन्छ ? .... दिन

३५ फलफुल खानेदिनमा, दिनको कतिपटक फलफुल खानुहुन्छ ? .... पटक

३६ साधारणतह, एक हप्तामा तपाई कति दिन हरियो सागपात / हरियो तरकारी खानुहुन्छ ? .... दिन

३७ सागपात / हरियो तरकारी खानेदिनमा, एक दिनमा कतिपटक सागपात / हरियो तरकारी खानुहुन्छ ? .... पटक

शारिरिक गतिविधिहरु

३८ विगत १ महिनामा, तपाईल नियमित काम बाहेक, अन्य कुनै शारिरिक व्यायाम / गतिविधिमा भाग लिनुभएको छ ?

1 छ	2 छैन

३९ यदि छ भने, कस्तो खालको शारीरिक व्यायाम भाग लिनुभएको छ ? ....

४० विगत १ महिनामा, महिनाको कति दिन भाग लिनुभएको छ ? .... दिन

४१ विगत १ महिनामा, शारिरीक एक्सरसाइज गर्ने दिन कति मिनेटसम्म गर्नुभयो / गर्नुहुन्छ ? ..... मिनेट

स्क्रीनिंग

४२ तपाई कहिले क्यान्सर छ कि छैन भनेर, स्वास्थ्य जाँच गराउन जानुभएको छ ?

1 छ	2 छैन

म्यामोग्राफी भनेको स्तनमा टिउमर छ कि छैन भनेर एक्सरे गरिने जाँच हो ।

कोलोनोस्कपी भनेको पेट तथा आन्दामा कुनै पकारको खरावी वा असामान्यतया पत्ता लगाउन गरिने जाँच हो ।

पिएसए भनेको प्ोस्टेटको क्यान्सर पत्ता लगाउन गरिने जाँच हो ।

प्याप स्मीयर टेस्ट भनेको पाठेघरको क्यान्सर पत्ता लगाउन गरिने जाँच हो ।

४३ तलका मध्ये कुनै जाँच गराउनु भएको छ ?

स्कीनिङ	छ	छैन
१ म्यामोग्राफी, विगत दुई वर्षसम्म ?	1	2
२ कोलोनोस्कपी, विगत १० वर्षमा ?	1	2
३ पिएसए विगत, दुई वर्षमा ?	1	2
४ प्याप स्मीयर, विगत तीन वर्षमा ?	1	2

सहयोगको लागि धन्यवाद
